# Awareness and knowledge of familial breast and ovarian cancer among German general practice patients

**DOI:** 10.1002/jgc4.70105

**Published:** 2025-08-22

**Authors:** Diana Guertler, Ann‐Kristin Reinhard, Sabina Ulbricht, Jean‐François Chenot, Ute Felbor, Susanne Wurm

**Affiliations:** ^1^ Department of Methods of Community Medicine, Institute for Community Medicine University Medicine Greifswald Greifswald Germany; ^2^ DZHK (German Centre for Cardiovascular Research), Partner Site Greifswald Greifswald Germany; ^3^ Department of Prevention Research and Social Medicine, Institute for Community Medicine University Medicine Greifswald Greifswald Germany; ^4^ Department of SHIP/KEF, Institute for Community Medicine University Medicine Greifswald Greifswald Germany; ^5^ Department of General Practice, Institute for Community Medicine University Medicine Greifswald Greifswald Germany; ^6^ Department of Human Genetics, University Medicine Greifswald, Interfaculty Institute of Genetics and Functional Genomics University of Greifswald Greifswald Germany

**Keywords:** awareness, breast and ovarian cancer, familial cancer, general public, knowledge

## Abstract

The aim of this cross‐sectional study was to describe the awareness and knowledge of familial breast and ovarian cancer among German males and females. Participants were patients ≥18 years from six general practices in Mecklenburg‐Western Pomerania. Between April 26 and July 11, 2024, all patients in the waiting room of the practices were systematically approached by a study assistant and invited to an anonymous self‐administered survey on familial cancer prevention and health behaviors. A total of 479 (67.0%) patients participated, and 437 with complete outcome data were analyzed (mean age = 54.0, SD = 16.6; males 34.6%). Chi‐squared and *t*‐tests were used to examine potential disparities in awareness and knowledge of familial breast and ovarian cancer by gender, age, community size, education level, and cancer history. A large proportion (75.5%) of the participants had heard of hereditary breast or ovarian cancer, and 58.4% had heard of genetic counseling. Awareness of the certified center for familial breast and ovarian cancer in Greifswald (32.5%) and genetic testing for breast and ovarian cancer (39.8%) was lower. On average, 43% of the knowledge questions were correctly answered: largest knowledge gaps concerned the heritability of cancer through fathers and the frequency of gene mutations. Awareness and knowledge levels varied by gender, age, education, and cancer history. Low awareness of genetic counseling and testing may prevent at‐risk families from seeking early interventions. Dissemination of knowledge to the general public should focus particularly on individuals with lower education, as they are currently the least informed.

**What is known about this topic**

Inherited gene mutations contribute to the risk of many cancers, including breast and ovarian cancer. Public awareness and knowledge of familial cancer risks are essential for early detection and prevention.

**What this paper adds to the topic**

As recent data for Germany are lacking, this study examines awareness and knowledge of familial breast and ovarian cancer among German males and females and explores potential disparities by gender, age, community size, education, and cancer history.

## 1 INTRODUCTION

Many cancers including breast and ovarian cancer can be associated with inherited gene mutations (Breast Cancer Association Consortium et al., 2021; Momenimovahed et al., 2019; Rolfes et al., 2022). Enhancing public awareness of familial cancer risks is essential for improving early detection and prevention strategies. Targeted public health initiatives, such as media campaigns, can help to fill knowledge gaps and encourage individuals to review their family history or seek genetic counseling (Wakefield et al., 2010).

General public awareness of genetic risk factors of diseases is limited in both males and females worldwide, with only 60% being aware of genetic risk factors for breast cancer (e.g., Smerecnik et al., 2008). In a recent study from Palestine, only half of the 5411 females surveyed named breast or ovarian cancer in a close relative as a risk factor for developing ovarian cancer (Elshami et al., 2022). Awareness of genetic counseling and testing for hereditary cancer is also insufficient in the public (Hann et al., 2017). For example, in a US study of 940 residents of Pennsylvania and New Jersey, only 52% were aware of genetic testing for cancer (Giri et al., 2021). Among 203 residents of the rural Midwest of the United States, only 47% had heard of the term genetic counseling (Riesgraf et al., 2015).

Data suggest that awareness and knowledge may be unequally distributed across the population (Alghuson et al., 2022; Altiner et al., 2023; Elshami et al., 2022; Eum et al., 2018; Farsi et al., 2020; Giri et al., 2021; Krakow et al., 2018; Rose et al., 2005; Smerecnik et al., 2008; Wang et al., 2022), with better education, younger age, female gender, having a family history of cancer, and living in urban areas being associated with higher awareness and knowledge of genetic cancers, genetic disease risk factors, and genetic testing.

Regarding the German general public, a study in 2001/2002 investigated knowledge about familial breast cancer (Barth et al., 2004). However, the sample only included females from Freiburg. The 469 females aged 18–65 answered on average 2.4 out of 4 knowledge questions about familial breast cancer correctly.

In addition, 108 German males and females seeking advice at a center for familial breast and ovarian cancer were asked about their knowledge on genetic counseling and testing prior to their initial consultation (Weber, 2007). Most (73%) had never heard or had only heard a little about “breast cancer” genes beforehand. Many also knew little or nothing about genetic counseling (59%), genetic testing (59%) and the process and duration of genetic testing (82%).

However, these studies date back over one or two decades. They were conducted before genetic testing became more widely available, and before public awareness of inherited cancer risks increased dramatically following media attention in 2013 (Troiano et al., 2017). Therefore, the aim of this study was to describe the current awareness and knowledge of familial breast and ovarian cancer among German males and females. We aimed to investigate potential disparities in awareness and knowledge based on gender, age, community size, education level, and cancer history.

## 2 METHODS

### 2.1 Study population and procedures

This was a cross‐sectional study among patients ≥18 years visiting general practices in Mecklenburg‐Western Pomerania. Medical practices are highly suitable for reaching the population, as 88% of adults in Germany have contact with a general practitioner at least once a year (Prütz & Rommel, 2017). The only certified center for familial breast and ovarian cancer in Mecklenburg‐Western Pomerania is located in Greifswald (Felbor et al., 2022). We therefore randomly selected practices outside Greifswald, stratified by area (*n* = 5 practices from Stralsund, a city of about 60,000 inhabitants and about 35 km distance from Greifswald; and *n* = 7 practices from smaller towns and villages within a distance of 15–45 km from Greifswald), and contacted them by letter followed by phone calls. We aimed to recruit an equal number of practices per stratum, with a maximum recruitment period of 2 weeks per participating practice. Of the practices contacted, *n* = 6 participated (*n* = 3 from each stratum) and one declined. The other five practices needed more time to consult practice partners or were on holiday. We did not further encourage these practices to participate, as a sufficient number of practices had already been recruited to reach the target sample size of 424 patients. All patients who were encountered in the waiting room of the participating practices were systematically approached by a study assistant and invited to an anonymous self‐administered survey on familial cancer prevention and health behaviors. The survey was administered on tablet PCs and lasted about 10 min. Patients were provided with reading glasses and/or a paper‐based survey if needed. Individuals who were unable to participate in the survey were excluded: too ill, hearing or visual impairment, cognitive impairment, insufficient knowledge of the German language, and patients with a caregiver. No incentive was provided for participation in the study. Verbal informed consent was obtained from participants. Recruitment took place between April 26 and July 11, 2024. Practices were free to choose specific days or times for recruitment. In total, we recruited during 32 morning and 8 afternoon consultation times (a total of 163 h). Interested patients were given a flyer from the certified center for familial breast and ovarian cancer in Greifswald, which is one of 23 academic centers in the German Consortium Hereditary Breast and Ovarian Cancer. The study was approved by the ethical review board of the University Medicine Greifswald (BB 013/24; 12.02.2024).

The authors assert that all procedures contributing to this work comply with the ethical standards of the relevant national and institutional committees on human experimentation and with the Declaration of Helsinki.

### 2.2 Measures

To test procedures and acceptance of the survey questions, a 4‐h pretest was conducted in one rural practice. A total of 19 patients (participation rate: 82.6%) between 18 and 80 years participated and were instructed to highlight questions difficult to understand. Only minor changes had to be made to the pretested survey questions based on the participants' feedback. The final survey questionnaire in German language is attached in Data S1.

#### 2.2.1 Awareness

Hereditary cancer was introduced as follows: “Next, we would like to ask you a few questions about cancer. These can also be hereditary. This means that their development is based on changes in genes that are passed on from generation to generation within a family. This is sometimes the case with breast or ovarian cancer, for example.” Then, yes/no questions were used to assess whether respondents had ever heard of hereditary breast or ovarian cancer, genetic counseling, the center for familial breast and ovarian cancer in Greifswald, or genetic testing for breast and ovarian cancer. In addition, the respondents were asked whether their doctor had ever raised the issue of hereditary breast cancer or hereditary ovarian cancer with them.

#### 2.2.2 Knowledge

To assess knowledge of familial breast and ovarian cancer, items from an established instrument (Lerman et al., 1996) were adapted. Adaptations of the instrument have already been used in population‐based surveys (Amin et al., 2012; Barth et al., 2004; Rose et al., 2005). Redundant and inappropriate items for the general population were not included in the current survey, resulting in a total of six items being used (Data S2). The number of correctly answered knowledge items was calculated.

#### 2.2.3 Socio‐demographics

Age, gender (male, female, and other), community size classes of the participant's place of residence (Behrens et al., 2019), educational level, partnership status “Are you currently living in a partnership with someone in your household? (yes/no),” and the number of biological children were assessed. Education level was assessed using a nine‐category system ranging from attending a full‐time general education school to various levels of school‐leaving certificates, including secondary and higher education qualifications. Categories were then collapsed according to the usual years of schooling (<10 years of schooling, 10 years of schooling, and >10 years of schooling).

#### 2.2.4 Personal and family cancer history

Two questions were used: “Have you ever been diagnosed with cancer yourself?” and “Has anyone else in your family ever been diagnosed with cancer? Please think of all blood relatives including parents, siblings, children, grandparents, aunts, uncles, nephews and nieces.” After each of the questions, participants were instructed to select types of cancers that have occurred (breast cancer, ovarian cancer, bowel cancer, prostate cancer, other cancer(s), and don't know).

### 2.3 Sample size calculation

The primary outcome of this study was the number of correctly answered questions on familial breast and ovarian cancer knowledge (Lerman et al., 1996). We aimed to investigate potential group differences in knowledge. A sample size of *N* = 424 for a 2 × 2 design was calculated to detect a 10% difference using *t*‐tests (alpha = 0.05, power = 0.80), based on estimated standard deviation (Barth et al., 2004). Due to deviations in empirical standard deviation and unequal subgroup sizes, we ensured that each subgroup had a sufficient sample size to identify the 10% difference (Data S2).

### 2.4 Statistical analyses

#### 2.4.1 Awareness

The percentage of individuals aware of the existence of hereditary breast or ovarian cancer, genetic counseling, the center for familial breast and ovarian cancer in Greifswald, and genetic testing was reported as well as the percentage of individuals reporting that their doctor had ever raised the issue of hereditary breast cancer or hereditary ovarian cancer with them. Chi‐squared tests were used to examine potential group differences according to gender, age, community size, education level, and cancer history.

#### 2.4.2 Knowledge

Mean and standard deviation of the number of knowledge items answered correctly (Lerman et al., 1996), and the percentage of individuals with correct answers on an item‐by‐item basis was reported. Group comparisons were made using *t*‐tests to test the hypothesis that knowledge differs depending on gender, age, community size, education level, and cancer history. Grouping variables were dichotomized based on examination of the distribution of the variables, either by identifying the point at which knowledge showed a significant change or by using a cut‐off that ensured similar sample sizes in the subgroups (e.g., piecewise regression (Muggeo, 2008) indicated a change point at 80 years, but due to insufficient patients over 80 years, we divided groups based on the median).

#### 2.4.3 Cohen's *d* measured effect sizes for *t*‐tests

Values above |0.2| indicate small, above |0.5| moderate, and above |0.8| large effects (Cohen, 1988). For chi‐square tests, Cramer's *V* was used: values above 0.1 indicate small effects, above 0.3 moderate, and above 0.5 large in two‐column tables (Cohen, 1988).

## 3 RESULTS

### 3.1 Participation and sample characteristics

Of the 715 eligible patients, 67.0% (*n* = 479) participated in the survey. The flow of participants is shown in Data S2. Survey participants were younger (odds ratio [OR] = 0.97; confidence interval [CI] 0.95–0.98; *p* < 0.001) and more likely to be female (OR = 2.17; CI 1.58–2.99; *p* < 0.001) than non‐participants. The participation rate was 56.9% for males and 74.2% for females. The participation rate varied between 57.6% and 78.3% among the participating general practices. Acceptance of the digital questionnaire was high, with only 7.7% (*n* = 37) requesting a paper–pencil questionnaire. Those preferring the paper–pencil questionnaire were significantly older (OR = 1.16; CI 1.11–1.21; *p* < 0.001) than digital questionnaire users. Gender had no significant influence on preference (OR = 0.62; CI 0.31–1.21; *p* = 0.161).

Those with complete survey data on knowledge and socio‐demographics (*n* = 437) were included in the following analyses. Characteristics of the study population are shown in Table 1. Participants were aged between 18 and 92 years (Mean [*M*] = 54.0; Standard Deviation [SD] = 16.6). The proportion of males was 34.6%.

**TABLE 1:** Demographic characteristics of the study sample.

Characteristics	Total (*n* = 437)	Males (*n* = 151)	Females (*n* = 286)
Age, *M* (SD)	54.0 (16.6)	53.5 (16.9)	54.3 (16.4)
Community size, *n* (%)			
<2000	139 (31.8)	51 (33.8)	88 (30.8)
2000 to under 5000	29 (6.6)	11 (7.3)	18 (6.3)
5000 to under 20,000	71 (16.3)	21 (13.9)	50 (17.5)
20,000 to under 50,000	49 (11.2)	19 (12.6)	30 (10.5)
50,000 to under 100,000	139 (31.8)	45 (29.8)	94 (32.9)
100,000 to under 500,000	6 (1.4)	3 (2.0)	3 (1.1)
≥ 500,000	4 (0.9)	1 (0.7)	3 (1.1)
Education level, *n* (%)			
<10 years of schooling	72 (16.5)	35 (23.2)	37 (12.9)
10 years of schooling	207 (47.4)	60 (39.7)	147 (51.4)
>10 years of schooling	158 (36.2)	56 (37.1)	102 (35.7)
Living in a partnership, *n* (%)	301 (68.9)	101 (66.9)	200 (69.9)
Personal history of cancer, *n* (%)	55 (12.6)	21 (13.9)	34 (11.9)
Breast cancer	11 (2.5)	0 (0.0)	11 (3.8)
Ovarian cancer	2 (0.5)	–	2 (0.7)
Colon cancer	4 (0.9)	1 (0.7)	3 (1.0)
Prostate cancer	3 (0.7)	3 (2.0)	–
Other cancers	34 (7.8)	17 (11.3)	17 (5.9)
Don't know/ nothing stated	3 (0.7)	1 (0.7)	2 (0.7)
Family history of cancer, *n* (%)	311 (71.2)	108 (71.5)	203 (71.0)
Breast cancer	93 (21.3)	21 (13.9)	72 (25.2)
Ovarian cancer	22 (5.0)	5 (3.3)	17 (5.9)
Colon cancer	71 (16.2)	23 (15.2)	48 (16.8)
Prostate cancer	41 (9.4)	21 (13.9)	20 (7.0)
Other cancers	177 (40.5)	58 (38.4)	119 (41.6)
Don't know/ nothing stated	10 (2.3)	7 (4.6)	3 (1.0)
Number of biological children, *M* (SD)	1.5 (1.1)	1.4 (1.2)	1.5 (1.1)
None, *n* (%)	112 (25.6)	50 (33.1)	62 (21.7)

*Note*: *M*, mean; SD, standard deviation.

### 3.2 Awareness

A large proportion (75.5%) of the participants had heard of hereditary breast or ovarian cancer, and 58.4% had heard of genetic counseling (Table 2). Awareness of the center for familial breast and ovarian cancer in Greifswald (32.5%) and genetic testing for breast and ovarian cancer (39.8%) was lower. None of the awareness measures differed significantly by community size. However, females were significantly more aware of hereditary breast or ovarian cancer compared with males (79.4% vs. 68.2%; *p* = 0.010, *V* = 0.12). Similarly, awareness of genetic testing for breast and ovarian cancer was higher among females than males (45.1% vs. 29.8%; *p* = 0.002, *V* = 0.15).

**TABLE 2:** Differences in awareness measures based on gender, age, community size, education level, and cancer history.

	*n*	Hereditary breast or ovarian cancer (%)	Genetic counseling (%)^a^	Center for familial breast and ovarian cancer in Greifswald (%)^b^	Genetic test for breast or ovarian cancer (%)^a^
Total	437	75.5	58.4	32.5	39.8
Gender		***p* = 0.010, V = 0.12**	*p* = 0.241, V = 0.06	*p* = 0.296, V = 0.05	***p* = 0.002, V = 0.15**
Male	151	68.2	54.3	29.1	29.8
Female	286	79.4	60.5	34.3	45.1
Age		*p* = 0.302, V = 0.05	*p* = 0.807, V = 0.01	***p* = 0.002, V = 0.15**	*p* = 0.695, V = 0.02
≤55 years	215	77.7	59.1	25.6	39.1
>55 years	222	73.4	57.7	39.2	40.5
Community size		*p* = 0.670, V = 0.02	*p* = 0.463, V = 0.04	*p* = 0.165, V = 0.07	*p* = 0.968, V = 0.002
≤5000	168	74.4	56.0	36.3	39.9
>5000	269	76.2	59.9	30.1	39.8
Education level		*p* = 0.393, V = 0.04	*p* = 0.520, V = 0.03	***p* = 0.031, V = 0.10**	*p* = 0.744, V = 0.02
≤10 years of schooling	279	74.2	57.4	36.2	39.4
>10 years of schooling	158	77.9	60.1	26.0	40.5
Personal or family history^c^		***p* < 0.001, V = 0.19**	***p* = 0.004, V = 0.14**	***p* = 0.015, V = 0.12**	***p* = 0.002, V = 0.15**
No	251	68.5	52.6	27.9	33.5
Yes	186	85.0	66.1	38.7	48.4

^a^ One missing value.

^b^ Two missing values.

^c^ Of breast, ovarian, colon, or prostate cancer.

V, Cramer's V; Displayed are results from chi‐square tests; comparisons with Cramer's *V* ≥ 0.10 are displayed as bold.

Those older than 55 years were more aware of the center for familial breast and ovarian cancer in Greifswald than those aged 55 or younger (39.2% vs. 25.5%; *p* = 0.002, *V* = 0.15). Individuals with 10 or fewer years of schooling also showed higher awareness of the center compared to those with more than 10 years of schooling (36.2% vs. 26.0%; *p* = 0.031, *V* = 0.10). Furthermore, those with a personal or family history of breast, ovarian, colon, or prostate cancer had significantly higher awareness for all four measures compared with those with no or other cancer history.

In addition, 29.8% of respondents reported that their doctor had raised the issue of hereditary breast cancer or hereditary ovarian cancer with them before. This rate did not differ significantly by age (*p* = 0.900, *V* = 0.01), community size (*p* = 0.578, *V* = 0.03), or education level (*p* = 0.142, *V* = 0.07), but did differ by gender (4.6% of males vs. 43.0% of females; *p* < 0.001, *V* = 0.40) and personal or family history of breast, ovarian, colon, or prostate cancer (22.7% of those with no history vs. 39.3% of those with a history; *p* < 0.001, *V* = 0.18).

### 3.3 Knowledge

Descriptive statistics for the knowledge measure at item level are presented in Table 3. The percentage of participants answering correctly was highly dependent on the item (range from 5% to 70%), with many “don**'**t know” responses also recorded.

**TABLE 3:** Descriptive statistics for the knowledge measure on item level.

Items	% of participants answering		
	Correctly	Incorrectly	Don't know
A father can pass down an altered breast or ovarian cancer gene to his children (T)	26.1	28.2	45.8
Ovarian cancer is often only discovered when it has already spread (T)	39.4	31.8	28.8
Men cannot develop breast cancer (F)	69.8	13.7	16.5
All women who have an altered breast or ovarian cancer gene will get cancer (F)	52.0	8.0	40.1
About 1 in 10 women have an altered breast or ovarian cancer gene (F)	5.0	35.5	59.5
A woman with an altered breast or ovarian cancer gene has a higher risk of developing breast or ovarian cancer (T)	65.0	3.7	31.4

*Note*: F, false item; T, true item.

The mean number of correctly answered knowledge questions in the total sample was *M* = 2.6 (SD = 1.4, range 0–6). As can be seen in Table 4, the number of correctly answered knowledge questions did not differ significantly by gender (*p* = 0.263, *d* = 0.11) or by community size (*p* = 0.224, *d* = 0.12). Knowledge was significantly higher among younger participants (*p* = 0.010, *d* = −0.25) and those with higher education (*p* < 0.001, *d* = 0.43). A personal or family history of breast, ovarian, colon, or prostate cancer was associated with significantly higher knowledge among females (*p* = 0.007, *d* = 0.32), but not among males (*p* = 0.556, *d* = −0.10).

**TABLE 4:** Differences in knowledge based on gender, age, community size, education level and cancer history.

	Number of correctly answered knowledge questions, *M* (SD)		
	Total (*n* = 437)	Males (*n* = 151)	Females (*n* = 286)
Gender	*p* = 0.263, *d* = −0.11	–	–
Male	2.5 (1.5)		
Female	2.6 (1.3)		
Age	***p* = 0.010, *d* = −0.25**	***p* = 0.158, *d* = −0.23**	***p* = 0.031, *d* = −0.26**
≤55 years	2.7 (1.4)	2.6 (1.5)	2.8 (1.4)
>55 years	2.4 (1.3)	2.3 (1.4)	2.5 (1.3)
Community size	*p* = 0.224, *d* = 0.12	*p* = 0.809, *d* = 0.04	*p* = 0.189, *d* = 0.16
≤5000	2.5 (1.4)	2.4 (1.5)	2.5 (1.3)
>5000	2.6 (1.4)	2.5 (1.4)	2.7 (1.3)
Education level	***p* < 0.001, *d* = 0.43**	***p* = 0.017, *d* = 0.41**	***p* < 0.001, *d* = 0.44**
≤10 years of schooling	2.4 (1.3)	2.3 (1.4)	2.4 (1.3)
>10 years of schooling	2.9 (1.4)	2.8 (1.5)	3.0 (1.3)
Personal or family history^a^	*p* = 0.073, *d* = 0.17	*p* = 0.556, *d* = −0.10	***p* = 0.007, *d* = 0.32**
No	2.5 (1.4)	2.5 (1.4)	2.4 (1.3)
Yes	2.7 (1.4)	2.4 (1.5)	2.9 (1.3)

*Note*: Displayed are results from independent *t*‐tests. Comparisons with Cohen's *d* ≥ |0.20| are displayed as bold.

Abbreviations: *d*, Cohen's *d*; M, mean; SD, standard deviation.

^a^ Of breast, ovarian, colon, or prostate cancer.

## 4 DISCUSSION

The aim of this study was to describe the awareness and knowledge of familial breast and ovarian cancer among German general practice patients, focusing on disparities according to gender, age, community size, education level, and cancer history. The findings highlight several significant gaps in public understanding that have implications for early detection, management, and prevention of cancer.

A substantial portion of the sample had heard about hereditary breast or ovarian cancer, indicating a baseline awareness of these conditions. However, awareness of specific services—such as genetic counseling, testing, and the center for familial breast and ovarian cancer in Greifswald—was notably lower. This suggests a significant gap between general awareness and actionable knowledge, which is crucial for potential at‐risk families. Low awareness may be partly due to the relatively recent availability of genetic testing for breast and ovarian cancer: Although the institute of human genetics in Greifswald was founded in 1978 and offered genetic counseling and molecular diagnostics with a focus on monogenic metabolic disorders, a dedicated center for familial breast and ovarian cancer was only established in 2018 (Felbor et al., 2022).

The fact that, on average, only 43% of the knowledge questions were correctly answered demonstrates an overall lack of understanding about familial breast and ovarian cancer. The selection of knowledge questions and the gender and education of the participants may partly explain the discrepancy with the Freiburg study (Barth et al., 2004), in which females answered 60% of the questions correctly. However, the exclusion of items on the frequency of occurrence of breast or ovarian cancer genes, which was the item with the lowest correct answers in our study, may partially explain the discrepancy.

Interestingly, the majority of participants in our study were informed about the existence of male breast cancer and the heightened risk posed by genetic predispositions. This is significant, as awareness of male breast cancer is typically low (Altiner et al., 2023). However, only 50% of participants correctly understood that genetic predisposition does not equate to absolute cancer risk (Smerecnik et al., 2008), revealing an important misunderstanding that could affect decision‐making regarding screening and preventive actions.

The most pronounced knowledge gaps concerned the heritability of pathogenic variants in cancer genes through fathers and the frequency of gene mutations. Only 26% of participants knew that a father could pass on an altered breast or ovarian cancer gene, a figure far below the 66% reported in an earlier US study (Rose et al., 2005). This gap in understanding paternal transmission is problematic, as it may lead families to overlook crucial genetic risks. Furthermore, only 5% of the sample correctly disagreed with the statement that about 1 in 10 women carry an altered breast or ovarian cancer gene, suggesting that the majority overestimated the occurrence of such gene alterations. This misperception is consistent with findings from a Dutch study (De Vries et al., 2005), indicating a general public tendency to overestimate the hereditary causes of breast cancer. Such misconceptions could contribute to unnecessary anxiety. A further finding was that < 40% of the sample knew that ovarian cancer is often detected only after it has spread. This suggests a lack of understanding of the rapid progression of the disease and the critical role of genetic mutations in its pathomechanism: Pathogenic genetic variants are the most important known risk factor for ovarian cancer (Momenimovahed et al., 2019). Recognition of a genetic predisposition may lead to predictive genetic testing of healthy at‐risk relatives. If a known familial pathogenic variant in one of the breast and ovarian cancer risk genes is confirmed in a healthy at‐risk relative, risk‐reducing surgery may be discussed and possibly performed.

The results of the study also highlight significant disparities in awareness and knowledge. The role of factors such as gender, age, education, and personal cancer history points to the need for targeted public health strategies that address these inequities. Although males and females had similar overall knowledge scores, males were less aware of hereditary breast or ovarian cancer and genetic testing, consistent with previous findings (Giri et al., 2021; Rose et al., 2005). This disparity reflects a gender gap in perceived relevance: Males may not view themselves as stakeholders in hereditary breast and ovarian cancer risk, even though paternal inheritance can significantly contribute to cancer predisposition. Public health initiatives need to deconstruct the misconception that hereditary breast and ovarian cancers are exclusively female concerns, promoting more inclusive cancer prevention strategies.

Similarly, older participants and those with lower education levels had lower overall knowledge (Wang et al., 2022), but were more aware of the center for familial breast and ovarian cancer in Greifswald. The latter could reflect older individuals' longer exposure to local healthcare information or their personal experiences with cancer, that is likely to increase with age. It seems counter intuitive that individuals with lower educational levels were more aware of the center for familial breast and ovarian cancer in Greifswald. However, they may be more likely to engage with service‐oriented information that directly addresses their healthcare needs (e.g., “Where can I go for help?”) than knowledge‐oriented information, while those with higher levels of education may feel that they already have sufficient knowledge and therefore do not need additional counseling.

Additionally, individuals with a personal or family history of breast, ovarian, colon, or prostate cancer had higher awareness of hereditary breast or ovarian cancer and genetic testing, supporting previous results (Atkinson et al., 2017). However, cancer history was associated with higher knowledge only among females. This observation highlights the need for gender‐specific public health approaches.

Unexpectedly, community size was not significantly associated with awareness or knowledge, contrasting with previous research showing higher cancer knowledge and testing awareness in urban areas (Eum et al., 2018; Wang et al., 2022). This may be due to the geographic proximity of both rural and urban communities to Greifswald, minimizing healthcare awareness and knowledge disparities. Rural communities further away from Greifswald may still face significant awareness or knowledge gaps.

This study has several limitations that should be acknowledged. First, reliance on cross‐sectional and self‐reported data introduces potential biases, and we cannot be certain whether responders differ systematically from non‐responders in their awareness and knowledge. Second, the lower participation rate among males and geographic restriction to a specific area of Western Pomerania may have limited representativeness. For instance, the proportion of individuals with over 10 years of schooling was around 10% higher in the present study than in the regional average for Mecklenburg‐Western Pomerania (Statistisches Bundesamt, 2022). This also restricts the generalizability of the findings to the wider German population. Third, other factors, such as race/ethnicity, health status, occupation, income, and marital status, may also have influenced the results (Elshami et al., 2022; Eum et al., 2018; Farsi et al., 2020; Krakow et al., 2018; Wang et al., 2022). Fourth, the participating practices varied in their specialization (e.g., internal medicine, general medicine, or diabetology), additional services (e.g., sonography, skin screening, chiropractic treatment, or addiction medicine), and the languages in which consultations were offered. These differences may have influenced the sample characteristics and responses. However, due to the way patient data were collected (e.g., without identifiers linking cases to specific practices in order to protect privacy), it was not possible to conduct a retrospective analysis of these effects. Fifth, our sample may include individuals who had previously undergone genetic counseling or testing for hereditary breast and ovarian cancer, as this information was neither assessed in the survey nor used as an exclusion criterion. Sixth, we cannot exclude the possibility that the introductory definition of hereditary cancer influenced the participants' responses regarding awareness and knowledge. Although the text was designed to provide basic conceptual clarity, it may have introduced measurement bias. Seventh, age was dichotomized at the sample median to ensure sufficient group sizes for statistical analysis. However, piecewise regression revealed a potential shift in knowledge levels around the age of 80. Due to the small number of participants in this age range, we were unable to use this threshold, which could limit the sensitivity of age‐related comparisons. Eighth, we adapted a knowledge questionnaire that had previously been validated among male and female members of families with hereditary breast and ovarian cancer. However, we did not assess its validity for use within the general population.

Despite these limitations, the study has notable strengths. It employed random sampling within general practices and systematic patient recruitment, alongside a priori sample size calculations. Additionally, the inclusion of both genders and a broad age range enhances the generalizability of the findings.

## 5 CONCLUSIONS

The identified gaps in awareness and knowledge of hereditary breast and ovarian cancer have significant implications for public health. Low awareness of genetic counseling and testing may prevent at‐risk families from seeking early interventions. Dissemination of knowledge to the general public should focus particularly on individuals with lower education, as they are currently the least informed. Importantly, improving knowledge on paternal heritability is essential to help families recognize their risks. Genetic counseling in Germany is currently provided by specialist physicians in human genetics. Expanding their visibility and integrating them more strongly into public health initiatives could help to reduce gaps in genetic literacy. Increasing awareness among general practitioners may promote appropriate referrals for genetic counseling, since taking family history is part of the preventive services they regularly provide (Ong et al., 2022); only a small proportion of patients seek genetic counseling without referral (Schmidtke et al., 2020). Another way to improve public understanding of genetics, health behaviors, and cancer is to share basic information with at‐risk populations. This can be achieved through special campaigns, educational programs, and online tools that calculate a family's cancer risk.

## AUTHOR CONTRIBUTIONS

DG: Conceptualization, data curation, formal analysis, funding acquisition, investigation, methodology, project administration, software, validation, visualization, writing – original draft preparation. A‐KR: Data curation, methodology, software, validation. SU: Conceptualization, methodology, supervision, validation. J‐FC: Conceptualization, methodology, supervision, validation. UF: Conceptualization, methodology, supervision, validation. SW: Conceptualization, methodology, resources, supervision, validation. All authors contributed to the review and editing of the article and have approved the final article.

## FUNDING INFORMATION

This work was supported by the research networks Community Medicine, Molecular Medicine, GANI_MED and Digital Health Lab of the University Medicine Greifswald (grant number: FOVB‐2024‐04).

## CONFLICT OF INTEREST STATEMENT

The authors declare that there is no conflict of interest.

## ETHICS STATEMENT

Human studies and informed consent: The study was approved by the ethical review board of the University Medicine Greifswald (BB 013/24; 12.02.2024). The authors assert that all procedures contributing to this work comply with the ethical standards of the relevant national and institutional committees on human experimentation and with the Declaration of Helsinki. Informed consent was obtained from all individual participants included in the study.

## ANIMAL STUDIES

No nonhuman animal studies were carried out by the authors for this article.

## Supporting information


Data S1

Data S2

## Data Availability

The data that support the findings of this study are available from the corresponding author upon reasonable request.
