# Supplementary material for: Awareness and knowledge of familial breast and ovarian cancer among German general practice patients
Source: J Genet Couns. 2025 Aug 22;34(4):e70105. doi: 10.1002/jgc4.70105 (PMC12374076; doi:10.1002/jgc4.70105)
Supplement: Supplementary file 1 — Data S1 [file JGC4-34-0-s001.docx]

**
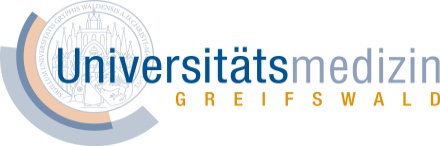
**

**Fragebogen:
Familiäre Krebsprävention und Gesundheitsverhalten**

Liebe Studienteilnehmer*innen,

vielen Dank für Ihre Bereitschaft, an dieser Studie teilzunehmen. Im Folgenden stellen wir Ihnen verschiedene Fragen zu Ihren persönlichen Verhaltensweisen und Ihren Einstellungen in Bezug auf die Prävention von Krebserkrankungen in der Familie.

Das Beantworten der Fragen dauert ca. 10 Minuten.

Die Daten werden **anonym** erhoben. Das bedeutet, es wird **weder Ihr Name, noch Ihr Geburtsdatum oder Ihre Anschrift** erfasst. Alle Ihre Angaben werden ausschließlich für wissenschaftliche Zwecke genutzt.

Das Ausfüllen des Fragebogens ist einfach:

- Kreuzen Sie einfach die jeweils zutreffende Antwortmöglichkeit in dem dafür vorgesehenen Kästchen an: 🗷
- Bei einigen Fragen bitten wir Sie um Zahlenangaben.
  Beispiel: Wie alt sind Sie? *18* Jahre
- Bitte überspringen Sie Fragen nur dann, wenn hinter dem von Ihnen angekreuzten Kästchen ein Hinweis gegeben wird.

Beispiel:

Bitte weiter mit Frage 9

**Vielen Dank für Ihre Teilnahme!**

**Projektkoordination:**

Dr. Diana Gürtler, Abteilung für Präventionsforschung und Sozialmedizin, Institut für Community Medicine, Universitätsmedizin Greifswald, diana.guertler@med.uni-greifswald.de

|  | **Bitte machen Sie zunächst ein paar Angaben zu Ihrer Person. Diese sind so allgemein, dass die Anonymität Ihrer Daten gewährleistet ist.** |
| --- | --- |
|  |  |
| **1.** | **Wie alt sind Sie?** |
|  | ______ Jahre |
| **2.** | **Welches Geschlecht haben Sie?** |
|  | □_1_ männlich □_2_ weiblich □_3_ anderes |
| **3.** | **Wie viele Einwohner hat Ihr Wohnort?** |
|  | □_1_ unter 2.000 Einwohner  □_2_ 2.000 bis unter 5.000 Einwohner  □_3_ 5.000 bis unter 20.000 Einwohner  □_4_ 20.000 bis unter 50.000 Einwohner  □_5_ 50.000 bis unter 100.000 Einwohner  □_6_ 100.000 bis unter 500.000 Einwohner  □_7_ 500.000 Einwohner und mehr |

| **Als nächstes möchten wir gern wissen, wie es Ihnen gesundheitlich geht.** | | |
| --- | --- | --- |
|  | | |
| **4.** | **Wie würden Sie Ihren Gesundheitszustand im Allgemeinen beschreiben?** | |
|  | □_1_ Ausgezeichnet  □_2_ Sehr gut  □_3_ Gut  □_4_ Weniger gut  □_5_ Schlecht | |
| **5.** | **Wie groß sind Sie?** | |
|  | ______ cm | |
| **6.** | **Wieviel wiegen Sie derzeit?** | |
|  | ______ kg | |
| **7.** | **Rauchen Sie aktuell?** | |
|  | □_1_ Ja, täglich  □_2_ Ja, gelegentlich | □_3_ Nein, ich habe früher geraucht  □_4_ Nein, ich habe nie geraucht |
| **8.** | **An wie vielen Tagen der letzten Woche waren Sie in Ihrer Freizeit insgesamt 30 Minuten oder länger körperlich aktiv, so dass Sie zumindest etwas stärker atmen mussten?**  *Beispiele für solche Aktivitäten sind Sport, Bewegung, Training sowie zügiges Gehen oder Fahrradfahren, entweder in der Freizeit oder um von Ort zu Ort zu gelangen.*  *Körperliche Aktivitäten im Haushalt oder im Rahmen Ihrer beruflichen Tätigkeit berücksichtigen Sie hingegen bitte nicht.* | |
|  | An ______ von 7 Tagen der letzten Woche | |
| **9.** | **An wie vielen Tagen haben Sie in den letzten 30 Tagen Alkohol getrunken?** | |
|  | An ______ von 30 Tagen  Bei 0 Tagen bitte weiter mit Frage 11 | |
| **10.** | Wenn Sie alkoholische Getränke zu sich nehmen, wie viele **Getränke** trinken Sie dann typischerweise **an einem Tag**?  *Ein alkoholisches Getränk entspricht z.B. 1 Bier 0,25-0,3l oder 1 Wein/Sekt 0,1-0,15l oder 1 doppelter Schnaps/Likör 4cl.* | |
|  | ______ alkoholische Getränke | |

| Wir möchten Ihnen als nächstes einige Fragen zu Krebserkrankungen stellen. Diese **können auch erblich bedingt sein**. D.h. ihre Entstehung beruht auf Veränderungen in den Genen, die innerhalb einer Familie von Generation zu Generation weitervererbt werden.  Zum Teil ist dies beispielsweise bei Brust- oder Eierstockkrebs der Fall. | |
| --- | --- |
|  |  |
| **11.** | **Haben Sie schon einmal von erblichem Brust- oder Eierstockkrebs gehört?**  *Bitte beachten Sie, wir meinen hier nicht Gebärmutterhalskrebs.* |
|  | □_1_ Ja □_2_ Nein |
| **12.** | **Wurden Sie schon einmal von Ihrem behandelnden Arzt auf das Thema erblicher Brust- oder Eierstockkrebs angesprochen?** |
|  | □_1_ Ja □_2_ Nein |

| **13.** | **Wussten Sie, dass es die Möglichkeit gibt, sich zu genetischen Fragen persönlich beraten zu lassen, z.B. im Zusammenhang mit der Familienplanung oder Häufungen von Erkrankungen in der Familie?** |
| --- | --- |
|  | □_1_ Ja □_2_ Nein |
| **14.** | **Wussten Sie, dass es in Greifswald ein Zentrum für familiären Brust- und Eierstockkrebs gibt, in dem man sich genetisch beraten lassen kann?** |
|  | □_1_ Ja □_2_ Nein |
| **15.** | **Mit einer genetischen Untersuchung (Gentest) lässt sich feststellen, ob jemand eine Veränderung in einem Brust- oder Eierstockkrebsgen hat.**  **Haben Sie schon einmal von solch einem Gentest gehört?** |
|  | □_1_ Ja □_2_ Nein |

| **16.** | **Geben Sie bitte an, ob folgende Aussagen Ihrer Meinung nach richtig oder falsch sind.** | | | |
| --- | --- | --- | --- | --- |
|  | | 1  Richtig | 2  Falsch | -1  Weiß nicht |
| Ein Vater kann ein verändertes Brust- oder Eierstockkrebsgen an seine Kinder weitergeben. | | □ | □ | □ |
| Eierstockkrebs wird oft erst entdeckt, wenn er sich bereits ausgebreitet hat. | | □ | □ | □ |
| Männer können nicht an Brustkrebs erkranken. | | □ | □ | □ |
| Alle Frauen, die ein verändertes Brust- oder Eierstockkrebsgen haben, erkranken an Krebs. | | □ | □ | □ |
| Etwa 1 von 10 Frauen hat ein verändertes Brust- oder Eierstockkrebsgen. | | □ | □ | □ |
| Eine Frau mit einem veränderten Brust- oder Eierstockkrebsgen hat ein höheres Risiko, an Brust- oder Eierstockkrebs zu erkranken. | | □ | □ | □ |
| Der eigene Lebensstil spielt bei der Entstehung von Brust- oder Eierstockkrebs eine wichtige Rolle. | | □ | □ | □ |

| **In den folgenden Fragen geht es ganz allgemein um die Durchführung genetischer Untersuchungen (Gentests).** |
| --- |
|  |

| **17.** | **Wie einverstanden sind Sie mit den folgenden Aussagen?** *Bitte kreuzen Sie in jeder Zeile an, was für Sie zutrifft!* | | | | | |
| --- | --- | --- | --- | --- | --- | --- |
|  | | 1  Überhaupt nicht einverstanden | 2  Eher nicht einverstanden | 3  Eher einverstanden | 4  Vollkommen einverstanden | -1  Weiß nicht |
| **Genetische Untersuchungen** | |  |  |  |  |  |
| … sollten allen zugänglich sein, die etwas über ihre Krankheitsrisiken erfahren möchten. | | □ | □ | □ | □ | □ |
| … sind akzeptabel, weil sie helfen können, die Kosten im Gesundheitswesen zu reduzieren. | | □ | □ | □ | □ | □ |
| … sind akzeptabel, weil alle das Recht haben, über ihre Gene Bescheid zu wissen und damit das eigene Leben und die Gesundheit beeinflussen können. | | □ | □ | □ | □ | □ |
| … sind nicht akzeptabel, weil die natürliche Ordnung respektiert werden sollte. | | □ | □ | □ | □ | □ |
| … sind nicht akzeptabel, weil die Ergebnisse zur Benachteiligung von Personen mit Veränderungen in Risikogenen führen könnten, z.B. in Form von höheren Versicherungsbeiträgen. | | □ | □ | □ | □ | □ |
| … sind nicht akzeptabel, weil dadurch Abtreibungen häufiger werden könnten. | | □ | □ | □ | □ | □ |

|  | **Die folgenden Fragen beziehen sich auf genetische Untersuchungen (Gentests), um festzustellen, ob jemand eine Genveränderung hat, die das Krebsrisiko erhöht.**  Es gibt verschiedene Gründe, die für die Durchführung von solchen genetischen Untersuchungen sprechen können. |
| --- | --- |

| **18.** | **Geben Sie bitte an, wie wichtig die folgenden Gründe für Sie persönlich sind.** | | | | | |
| --- | --- | --- | --- | --- | --- | --- |
|  | | 1  Nicht wichtig | 2 | 3 | 4 | 5  Sehr wichtig |
| Um etwas über das Krebsrisiko meiner Kinder zu erfahren. | | □ | □ | □ | □ | □ |
| Um zu wissen, ob ich häufiger zu Früherkennungsuntersuchungen gehen muss. | | □ | □ | □ | □ | □ |
| Um für die Zukunft zu planen. | | □ | □ | □ | □ | □ |
| Um Entscheidungen über medizinische Maßnahmen zu treffen, die mein Krebsrisiko senken. | | □ | □ | □ | □ | □ |
| Um beruhigt zu sein. | | □ | □ | □ | □ | □ |
| Um Entscheidungen in Bezug auf die Familienplanung zu treffen. | | □ | □ | □ | □ | □ |
| Damit ich meinen Lebensstil ändern kann. | | □ | □ | □ | □ | □ |

|  | Es gibt auch Gründe, die gegen die Durchführung von solchen genetischen Untersuchungen sprechen können. |
| --- | --- |

| **19.** | **Geben Sie bitte an, wie wichtig die folgenden Gründe für Sie persönlich sind.** | | | | | |
| --- | --- | --- | --- | --- | --- | --- |
|  | | 1  Nicht wichtig | 2 | 3 | 4 | 5  Sehr wichtig |
| Ich bin besorgt über mögliche negative Folgen für meine Versicherung. | | □ | □ | □ | □ | □ |
| Ich mache mir Sorgen über die Auswirkungen auf meine Familie, da z.B. Ängste oder Konflikte ausgelöst werden könnten. | | □ | □ | □ | □ | □ |
| Ich glaube nicht, dass Krebs verhindert werden kann. | | □ | □ | □ | □ | □ |
| Ich könnte es emotional nicht verkraften. | | □ | □ | □ | □ | □ |
| Die Untersuchungsergebnisse könnten ungenau sein. | | □ | □ | □ | □ | □ |
| Ich habe kein Vertrauen in die moderne Medizin. | | □ | □ | □ | □ | □ |
| Ich möchte nicht wissen, ob ich eine Genveränderung habe, die das Krebsrisiko erhöht. | | □ | □ | □ | □ | □ |

|  | **Die nächsten Fragen betreffen das Auftreten von Krebserkrankungen in Ihrer Familie.** |
| --- | --- |

| **20.** | **Wurde bei Ihnen selbst jemals eine Krebserkrankung diagnostiziert?**  Bitte weiter mit Frage 21 | |
| --- | --- | --- |
|  | □_1_ Ja  Bitte weiter mit Frage 22  □_2_ Nein | |
| **21.** | **Welche Art/en von Krebs war/en dies?** *Kreuzen Sie alles an, was zutrifft.* | |
|  | □ Brustkrebs  □ Eierstockkrebs  □ Darmkrebs | □ Prostatakrebs  □ andere Krebserkrankung(en)  □ Weiß nicht |

| **22.** | **Wurde bei jemandem anderen in Ihrer Familie jemals eine Krebserkrankung diagnostiziert?**  *Denken Sie hierbei bitte an alle mit Ihnen Blutsverwandten einschließlich Eltern, Geschwister, Kinder, Großeltern sowie Tanten, Onkel, Neffen und Nichten.* | |
| --- | --- | --- |
|  | □_1_ Ja  Bitte weiter mit Frage 24  Bitte weiter mit Frage 23  □_2_ Nein | |
| **23.** | **Welche Art/en von Krebs war/en dies?** *Kreuzen Sie alles an, was zutrifft.* | |
|  | □ Brustkrebs  □ Eierstockkrebs  □ Darmkrebs | □ Prostatakrebs  □ andere Krebserkrankung(en)  □ Weiß nicht |

|  | **Zum Abschluss möchten wir Ihnen noch drei Fragen zu Ihrer Person stellen.** |
| --- | --- |

| **24.** | **Leben Sie zurzeit mit einer Person aus Ihrem Haushalt in einer Partnerschaft?** |
| --- | --- |
|  | □_1_ Ja □_2_ Nein |
| **25.** | **Wie viele Kinder haben Sie?** *Bitte zählen Sie nur leibliche (blutsverwandte) Kinder.* |
|  | ______ Kinder |
| **26.** | **Welchen höchsten allgemeinbildenden Schulabschluss haben Sie?** |
|  | □_1_ Schüler/-in, besuche eine allgemeinbildende Vollzeitschule  □_2_ Von der Schule abgegangen ohne Schulabschluss  □_3_ Hauptschulabschluss (Volksschulabschluss) oder gleichwertiger Abschluss  □_4_ Polytechnische Oberschule der DDR mit Abschluss der 8. oder 9. Klasse  □_5_ Realschulabschluss (Mittlere Reife) oder gleichwertiger Abschluss  □_6_ Polytechnische Oberschule der DDR mit Abschluss der 10. Klasse  □_7_ Fachhochschulreife  □_8_ Abitur/Allgemeine oder fachgebundene Hochschulreife (Gymnasium bzw. EOS, auch EOS mit Lehre)  □_9_ Einen anderen Schulabschluss, und zwar ___________________________ |

**Vielen Dank für Ihre Teilnahme!**

**In Kürze wird unsere Studienmitarbeiterin wieder für Sie da sein und den Fragebogen abholen.**
