# Supplementary material for: Awareness and knowledge of familial breast and ovarian cancer among German general practice patients
Source: J Genet Couns. 2025 Aug 22;34(4):e70105. doi: 10.1002/jgc4.70105 (PMC12374076; doi:10.1002/jgc4.70105)
Supplement: Supplementary file 2 — Data S2 [file JGC4-34-0-s002.docx]

**Online Resource 2. Supplementary data**

**Table S1** Original and modified knowledge questions used in this study.

| Questions according to Lerman (1996) ^a^ | Modified questions used in this study (German language) | Modified questions used in this study  (English translation) |
| --- | --- | --- |
| True items |  |  |
| A father can pass down an altered BRCA1 gene to his daughters. | Ein Vater kann ein verändertes Brust- oder Eierstockkrebsgen an seine Kinder weitergeben. ^b^ | A father can pass down an altered breast or ovarian cancer gene to his children. |
| A woman who doesn't have an altered BRCA1 gene can still get cancer. | *Not included – redundant with following item* | **-** |
| A woman with an altered BRCA 1 gene has a high risk of ovarian cancer. | Eine Frau mit einem veränderten Brust- oder Eierstockkrebsgen hat ein höheres Risiko, an Brust- oder Eierstockkrebs zu erkranken. ^2^ | A woman with an altered breast or ovarian cancer gene has a higher risk of developing breast or ovarian cancer. |
| A woman who has a sister with an altered BRCA1 gene has a 50% chance of having an altered gene herself. | *Not included – not appropriate for the general population* | **-** |
| Tests for ovarian cancer often do not detect a tumor until it has spread. | Eierstockkrebs wird oft erst entdeckt, wenn er sich bereits ausgebreitet hat. | Ovarian cancer is often only discovered when it has already spread. |
| There are many different genes that cause cancer. | *Not included* | **-** |
| False items |  |  |
| All women who have an altered BRCA1 gene will get cancer. | Alle Frauen, die ein verändertes Brust- oder Eierstockkrebsgen haben, erkranken an Krebs. ^2^ | All women who have an altered breast or ovarian cancer gene will get cancer. |
| A woman who gets breast cancer at age 70 years is more likely to have an altered BRCA 1 gene than a woman who gets breast cancer at age 40 years. | *Not included – not appropriate for the general population* | **-** |
| Having one's ovaries removed will definitely prevent cancer. | Männer können nicht an Brustkrebs erkranken. ^c^ | Men cannot develop breast cancer. |
| The BRCA 1 gene causes about one half of all breast cancers. | *Not included – not appropriate for the general population* | **-** |
| About 1 in 10 women have an altered BRCA1 gene. | Etwa 1 von 10 Frauen hat ein verändertes Brust- oder Eierstockkrebsgen. | About 1 in 10 women have an altered breast or ovarian cancer gene. |

^a^ Lerman, C., Narod, S., Schulman, K., Hughes, C., Gomez-Caminero, A., Bonney, G., Gold, K., Trock, B., Main, D., & Lynch, J. (1996). BRCA1 testing in families with hereditary breast-ovarian cancer: a prospective study of patient decision making and outcomes. *JAMA*, *275*(24), 1885-1892. <https://doi.org/https://doi.org/10.1001/jama.1996.03530480027036>

^b^ Translation based on: Barth, J., Reitz, F., & Bengel, J. (2004). Einstellungen und Risikowahrnehmung bezüglich Brustkrebs und prädiktiver genetischer Brustkrebsdiagnostik - AttRisk Projektbericht [Attitudes and risk perception concerning breast cancer and predictive genetic testing - AttRisk project report]. Albert-Ludwigs-Universität Freiburg, Institut für Psychologie. Retrieved January 10, 2024, from <http://www.psychologie.uni-freiburg.de/einrichtungen/Reha/AbschlussberichtAttRisk.pdf>.

^c^ Asking males about ovarian removal may cause confusion or discomfort, thus we replaced this question with a more general statement.

**Table S2** Sample size calculation for two-sample t-tests with unequal group sample sizes.

| **Category** | **Gender**  (males vs. females) | **Age in years**  (≤ 55 vs. > 55) | **Community size**  (≤ 5000 vs. > 5000) | **Education: years of schooling**  (≤ 10 vs. > 10 years) | **Personal or family history** ^a^  (yes vs. no) |
| --- | --- | --- | --- | --- | --- |
| Empirical group ratio | 1:2 | 1:1 | 1:1.5 | 2:1 | 1.5:1 |
| Required n | **47:94** | **62:62** | **52:78** | **94:47** | **78:52** |
| Empirical n total | 151:286 | 215:222 | 168:269 | 279:158 | 251:186 |
| Empirical n in males | - | 75:76 | 62:89 | 95:56 | 91:60 |
| Empirical n in females | - | 140:146 | 106:180 | 184:102 | 160:126 |

Note. ^a^ of breast, ovarian, colon or prostate cancer; the sample size calculation included a mean difference of 0.7 to be detected by the test, the empirical standard deviation of knowledge (SD=1.38), alpha 0.05, and power 0.80.

**Figure S1** Flowchart of survey participants.

Already approached (n=19)

Registered consultations (n=825)

General practice patients (n=806)

Not eligible (n=91)

Not approached (n=6)

Age not ascertainable due to insufficient language skills (n=4) or illness (n=1)

Not willing to reveal age (n=29)

Age < 18 (n=6)

Hearing or visual impairment (n=4)

Insufficient language skills (n=29)

Cognitive impairment (n=1)

Too ill (n=11)

Eligible for participation (n=715)

Participated (n=479)

Complete data (n=424)

Incomplete data (n=55)

Technical problems (n=18) ^a^

Did not complete survey due to medical consultation or time constraints (n=19), or lack of satisfaction/interest (n=2)

Skipped paper-pencil items (n=13)

Invalid data ^b^ (n=3)

Refused participation (n=236)

^a^ Of the 479 survey participants, 442 participated via tablet and 37 asked for paper-pencil. Of the 442 tablet users, 24 experienced technical problems (sudden shutdown of the tablet). Of these 24 patients, n=5 did not repeat the survey. The other n=19 started the survey again (either on tablet n=5 or paper-pencil n=14), of which n=6 had complete data and n=13 did not answer all survey items);

^b^ Provided free-text answers for education could not be assigned to the predefined categories.
